# Expression of sialyl-Tn sugar antigen in bladder cancer cells affects response to *Bacillus Calmette Guérin* (BCG) and to oxidative damage

**DOI:** 10.18632/oncotarget.17138

**Published:** 2017-04-17

**Authors:** Paulo F. Severino, Mariana Silva, Mylene Carrascal, Nadia Malagolini, Mariella Chiricolo, Giulia Venturi, Annalisa Astolfi, Mariangela Catera, Paula A. Videira, Fabio Dall’Olio

**Affiliations:** ^1^ Centro de Estudos de Doenças Crónicas, CEDOC, NOVA Medical School, Faculdade de Ciências Médicas, Universidade NOVA de Lisboa, Lisboa, Portugal; ^2^ UCIBIO, Departamento Ciências da Vida, Faculdade de Ciências e Tecnologia, Universidade NOVA de Lisboa, Lisboa, Portugal; ^3^ Dipartimento di Medicina Specialistica, Diagnostica e Sperimentale, Sede di Patologia Generale, Università di Bologna, Bologna, Italy; ^4^ Centro Interdipartimentale Ricerche sul Cancro “Giorgio Prodi”, Università di Bologna, Bologna, Italy

**Keywords:** *Bacillus Calmette-Guérin*, bladder cancer, glycosylation, sialyl-Tn antigen, ST6GALNAC1 sialyltransferase

## Abstract

The sialyl-Tn (sTn) antigen is an *O*-linked carbohydrate chain aberrantly expressed in bladder cancer (BC), whose biosynthesis is mainly controlled by the sialyltransferase ST6GALNAC1. Treatment with *Bacillus Calmette-Guérin* (BCG) is the most effective adjuvant immunotherapy for superficial BC but one third of the patients fail to respond. A poorly understood correlation between the expression of sTn and BC patient's response to BCG was previously observed. By analyzing tumor tissues, we showed that patients with high ST6GALNAC1 and IL-6 mRNA expression were BCG responders. To investigate the role of sTn in BC cell biology and BCG response, we established the cell lines MCR_sTn_ and MCR_Nc_ by retroviral transduction of the BC cell line MCR with the *ST6GALNAC1* cDNA or with an empty vector, respectively. Compared with MCR_Nc_, BCG-stimulated MCR_sTn_ secreted higher levels of IL-6 and IL-8 and their secretome induced a stronger IL-6, IL-1β, and TNFα secretion by macrophages, suggesting the induction of a stronger inflammatory response. Transcriptomic analysis of MCR_Nc_ and MCR_sTn_ revealed that *ST6GALNAC1*/sTn expression modulates hundreds of genes towards a putative more malignant phenotype and down-regulates several genes maintaining genomic stability. Consistently, MCR_sTn_ cells displayed higher H_2_O_2_ sensitivity. In MCR_sTn_,, BCG challenge induced an increased expression of several regulatory non coding RNA genes. These results indicate that the expression of *ST6GALNAC1*/sTn improves the response to BCG therapy by inducing a stronger macrophage response and alters gene expression towards malignancy and genomic instability, increasing the sensitivity of BC cells to the oxidizing agents released by BCG.

## INTRODUCTION

Intravesical inoculation with *Bacillus Calmette-Guérin* (BCG) is an effective adjuvant therapy for treating non-muscle invasive bladder cancer (BC). However, a significant number of patients fails to respond. Although the precise mechanisms of its action remains uncertain, it is clear that BCG is internalized by cancer cells and induces a local inflammatory response, which is responsible for the anti-tumor effect [1–3]. Moreover, oxidizing agents released by BCG play a key role in the killing of BC cells [4]. A comprehensive understanding of how BCG modulates the immune system and induces phenotypic changes of BC could greatly improve patient's treatment.

**Figure 1:**
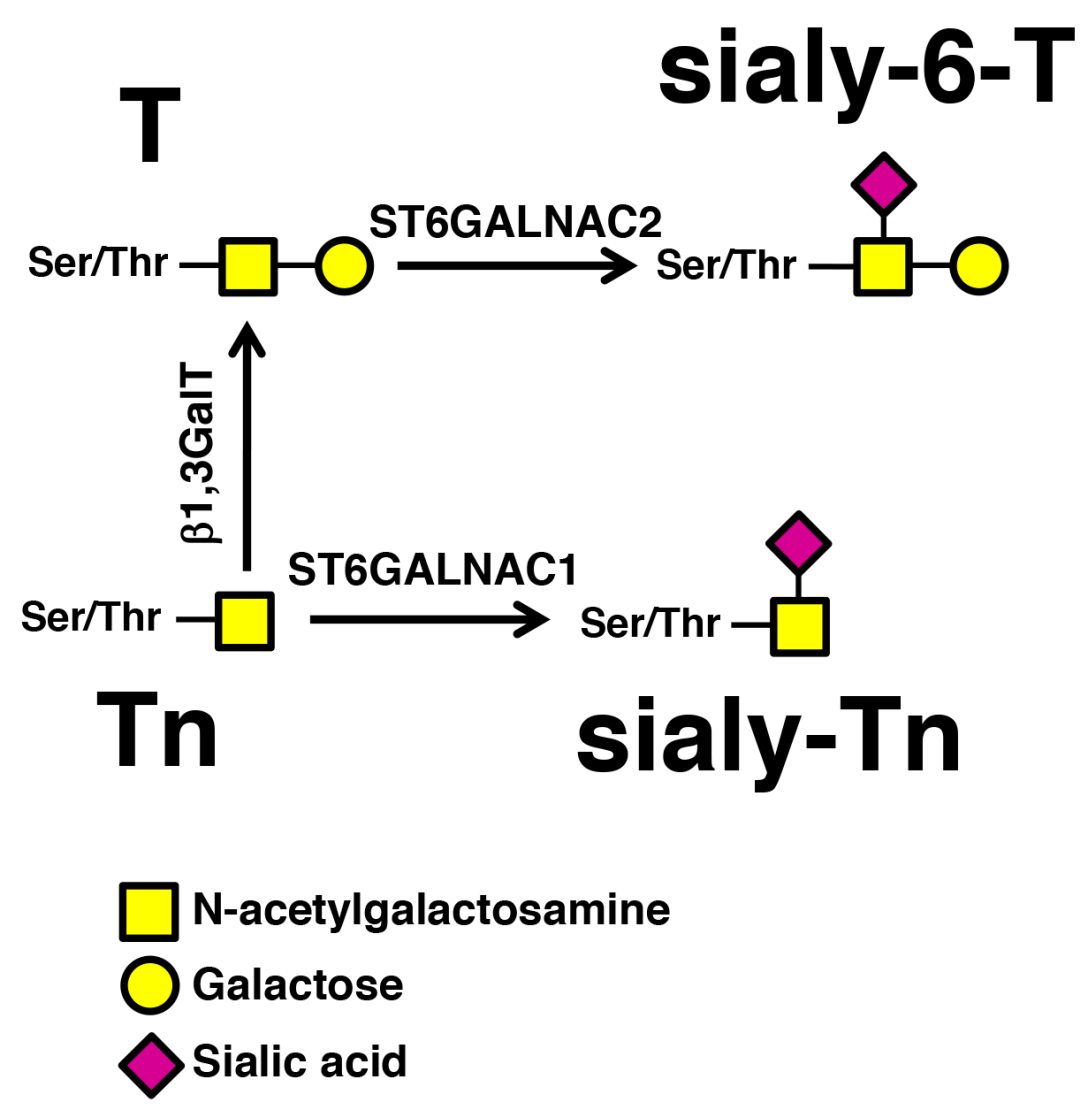
Biosynthesis of sTn by *ST6GALNAC1* The addition of α2,6-linked sialic acid on the Tn antigen (GalNAc-Ser/Thr), mediated by ST6GALNAC1 results in the biosynthesis of the sTn antigen, while the addition of α2,6-linked sialic acid on the T antigen (Galβ1,3GalNAc-Ser/Thr), resulting in the biosynthesis of the sialyl-6-T antigen, is catalyzed mainly by ST6GALNAC2.

Aberrant glycosylation of proteins is a common feature of several cancers [5, 6], including BC [7–10], and is often caused by the deregulation of glycosyltransferases, the enzymes which build the sugar portions of glycoconjugates. Several tumor-associated-antigens arise from deregulated glycosylation. Specifically, the tumor-associated-antigen sialyl-Tn (sTn) is a disaccharide *O*-glycosidically linked to serine or threonine, whose biosynthesis is mainly controlled by its cognate sialyltransferase ST6GALNAC1 (Figure 1) [9, 11, 12]. sTn is aberrantly expressed by a variety of human cancers, affecting their invasive potential [13–17]. Several reports have demonstrated that BC patients frequently overexpress this antigen and a correlation with an increased activity of ST6GALNAC1 has been consistently found [9, 13]. Furthermore, sTn-antigen-positive BC patients exhibit enhanced risk of recurrence and progression [9, 18]. Conversely, BC patients expressing the sTn and/or the related antigen sialyl-6-T (Figure 1) show a better response to BCG immunotherapy. Thus, sTn expression induces a more aggressive phenotype but a better clinical response to BCG, although the mechanisms underlying these biological effects remain to be elucidated.

In the present study, we compared *ST6GALNAC1* and *IL-6* gene expression in BC tissue from BCG responder and non-responder patients and showed that high ST6GALNAC1 mRNA expressing patients were BCG responders and had the tendency to express higher levels of IL-6 mRNA. In BC cells overexpressing or not *ST6GALNAC1* and its cognate sTn antigen, we studied cytokine secretion after BCG-challenge and their ability to stimulate inflammatory responses by macrophages. In addition, we characterized the transcriptome modulation induced by sTn expression and/or by BCG challenge in BC cells. Interestingly, we observed in sTn-expressing cells the down-regulation of several genes involved in maintaining genomic stability and, consistently, an increased sensitivity to the oxidizing activity of H_2_O_2_.

## RESULTS

### ST6GALNAC1 and IL-6 expression is associated with a better response to BCG

**Figure 2:**
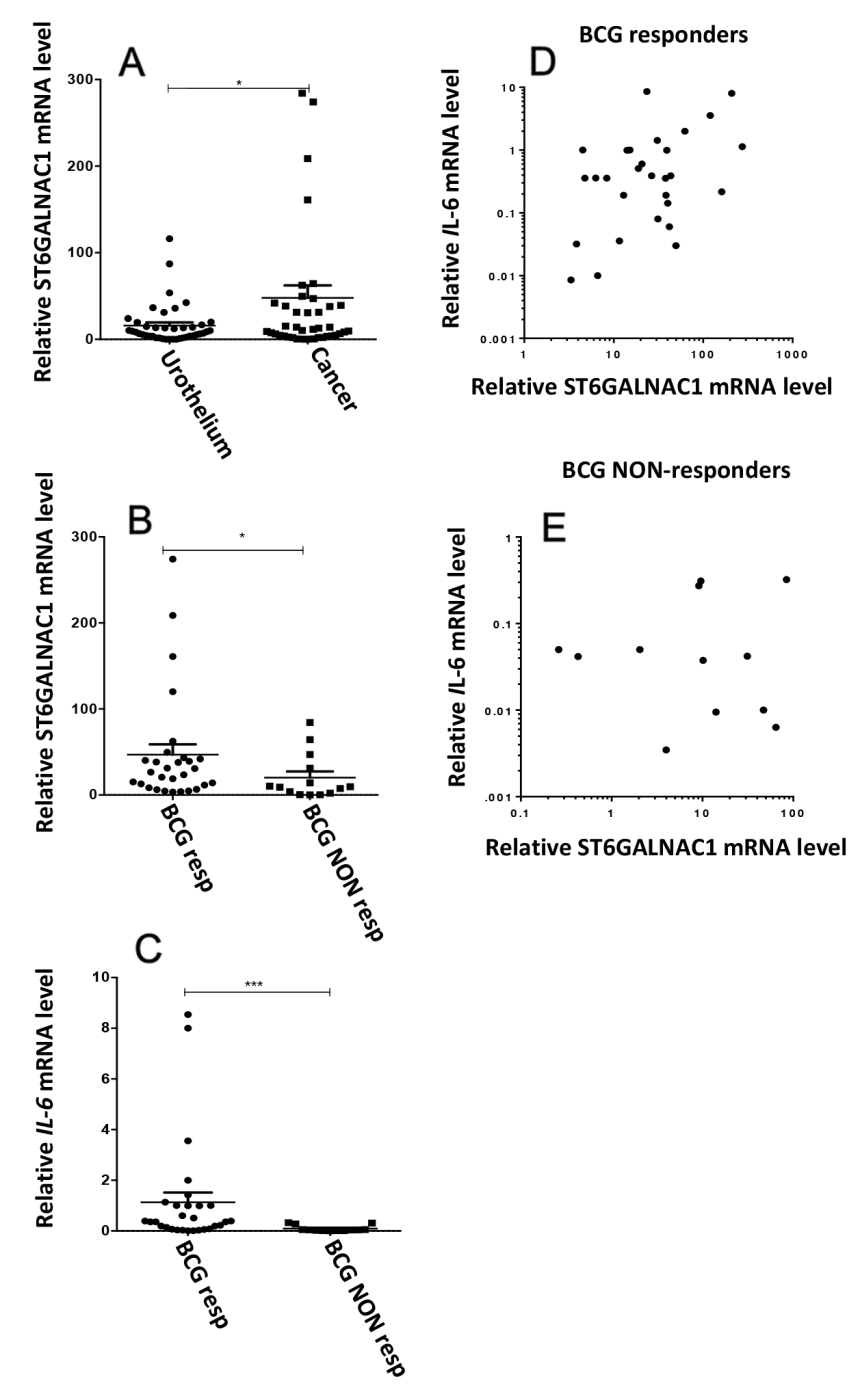
Gene expression analysis of surgical specimens of bladder cancers and normal urothelium Tissue specimens from 43 patients with nonmuscle-invasive bladder cancer eligible for BCG therapy, were collected, the RNA was extracted and the relative mRNA levels were analyzed by Real Time RT-PCR, as described in the Material and Methods section. Values indicate the number of mRNA molecules of *ST6GALNAC1* or *IL-6* genes per 1000 molecules of the reference gene (β-actin). **(A)** The expression of ST6GALNAC1 mRNA was compared in tumor tissue and matched normal urothelium and found to be significantly higher (*p*<0.05 according to Student's *t* test for paired samples) in tumor. **(B)** Patients were divided according to their follow up after BCG treatment. BCG Responders (Resp) were considered those without recurrence within a minimal period of 12 months following TURBT. BCG non responders (NON-Resp) were those that experienced disease recurrence within that period. High ST6GALNAC1 mRNA patients all belonged to the Responders group. **(C)** The expression of IL-6 mRNA was measured in the two groups and found to be higher in the Responders group. **(D** and **E)** correlation analysis indicated a significant (r= 0.375; p=0.04) relationship between ST6GALNAC1 and IL-6 mRNA expression in the responders group (D) but not in the non-responders group (E) (r= 0.281; p=0.33).

We analyzed the expression of *ST6GALNAC1* mRNA in matched tissues of normal urothelium and bladder cancer from 43 BC patients before BCG therapy. As shown in Figure 2A, the tumor tissue of a group of patients showed a very high ST6GALNAC1 mRNA expression. All these patients displayed good BCG response (Figure 2B), suggesting that ST6GALNAC1 mRNA overexpression identifies a group of patients with propensity to BCG response. In addition, the expression level of the IL-6 gene was also higher in BCG-responder patients (Figure 2C). Correlation analysis indicated a significant (r= 0.375; p=0.04) relationship between ST6GALNAC1 and IL-6 expression in the responders group (Figure 2D) but not in the non-responders group (r= 0.281; p=0.33), suggesting that patients with higher ST6GALNAC1 expression have the tendency to express higher levels of IL-6.

### sTn expression promotes pro-inflammatory cytokine secretion by BC cells

We studied *ST6GALNAC1*-transduced cell line MCR_sTn_, which comprises more than 95% of sTn-positive cells and expresses high levels of ST6GALNAC1 mRNA and enzyme activity, as previously reported [9]. Its respective negative control (MCR_Nc_) obtained by transduction with an empty vector expressed, like the wild type untransduced MCR cell line, negligible levels of the sTn antigen and of ST6GALNAC1 mRNA and enzyme activity [9, 13].

**Figure 3:**
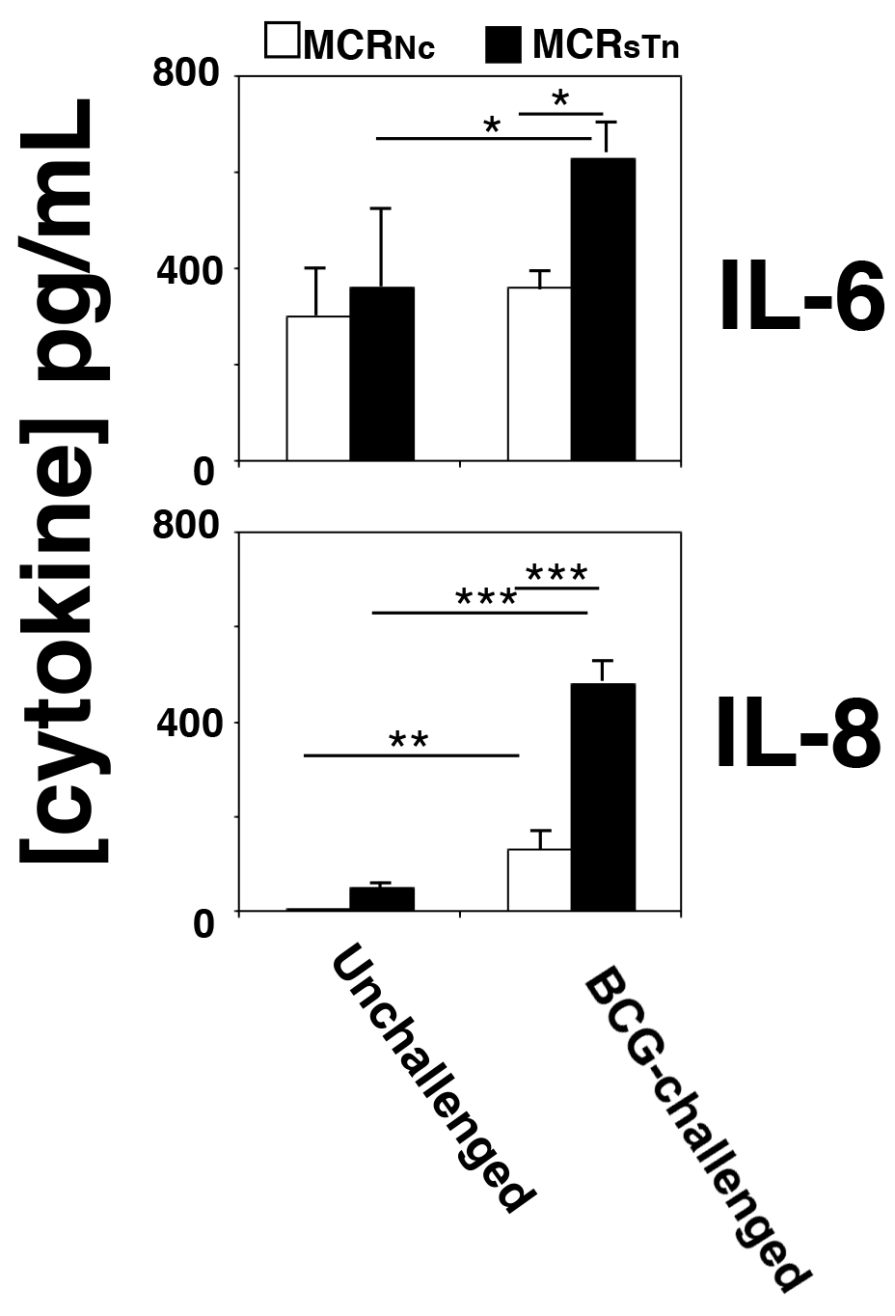
Cytokine secretion by BCG-challenged MCR cells MCR_Nc_ (white bars) and MCR_sTn_ (black bars) cells were challenged or not with BCG and the concentration of ten cytokines secreted was determined in their conditioned media as described in the Materials and Methods section. Only IL-6 and IL-8 were detectable and showed BCG modulation, mainly in MCR_sTn_ cells. Data are the mean ± SD of three experiments. ***p<0.0001; **p<0.001; *p<0.05, according to two ways ANOVA, followed by Tukey multiple comparison test. *p* values are reported in Supplementary Table 1.

To evaluate the effect of ST6GALNAC1 expression by BC cells on the induction of an inflammatory response triggered by BCG stimuli, the secretion of 10 cytokines (listed in Materials and Methods) was measured in conditioned media of MCR_Nc_ or MCR_sTn_, challenged or not with BCG. Preliminarily, the internalization of BCG by MCR_Nc_ or MCR_sTn_ was assessed by flow cytometry and found to be proportional to incubation time but not statistically different in the two cell populations for incubation times up to 6 h (data not shown), in agreement with previous results [10]. Of the cytokines tested, only IL-6 and IL-8 secretion was detectable. While both MCR_Nc_ and MCR_sTn_ cells natively produced high levels of IL-6, only MCRsTn cells significantly upregulated IL-6 secretion after BCG challenge (Figure 3 and Supplementary Table 1). In contrast, secretion of IL-8 was almost negligible in unchallenged cells but dramatically raised in both MCR_Nc_ and MCR_sTn_ cells after BCG challenge. Secretion of IL-8 after BCG challenge was also significantly higher in MCR_sTn_ cells than in MCR_Nc_ (Figure 3 and Supplementary Table 1). Altogether, these data suggest that the expression of ST6GALNAC1 and of its cognate sTn by BC promote the release of pro-inflammatory cytokines after BCG stimuli, crucial for the polarization and recruitment of inflammatory cells to BC sites.

### sTn expression by BC cells induces a stronger macrophage secretory response after BCG stimuli

**Figure 4:**
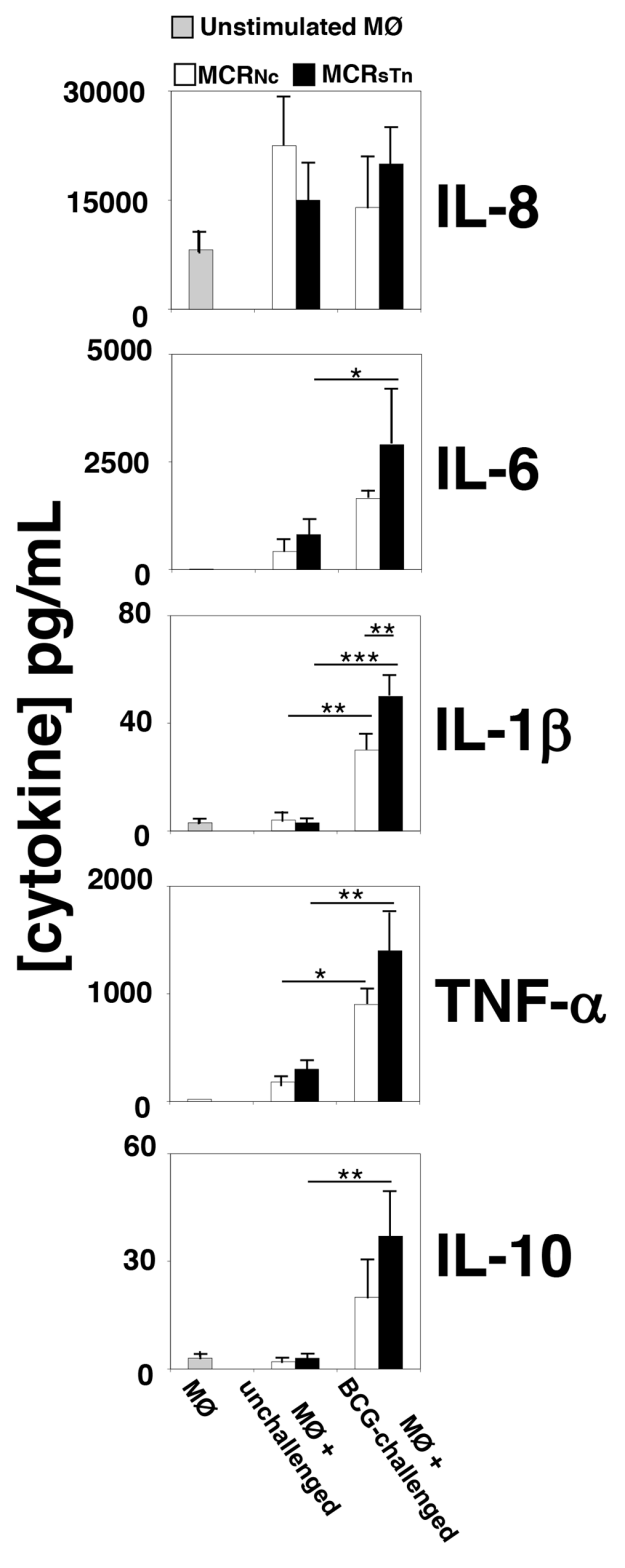
Cytokine secretion by macrophages treated with conditioned media of MCR cells The secretion of ten cytokines was measured in the culture media of unstimulated macrophages or of macrophages stimulated with the conditioned media of MCR_Nc_ (white bars) or MCR_sTn_ (black bars) either BCG-challenged or unchallenged, analyzed in Figure 3. Bars indicate the concentrations of cytokines detected in the conditioned medium of unstimulated macrophages (MØ, gray bars) or macrophages stimulated with conditioned media of unchallenged MRC_Nc_ or MCR_sTn_ cells (MØ + unchallenged) or stimulated with conditioned media of BCG-challenged MRC_Nc_ or MCR_sTn_ cells (MØ + BCG-challenged). Macrophage stimulation by unchallenged cells was negligible. With the exception of IL-8, the conditioned medium of BCG-challenged MCR_sTn_ cells (black bars) potentiated cytokine release by macrophages more than that of MCR_Nc_ (white bars). Data are the mean ± SD of 3 experiments. ***p<0.0001; **p<0.001; *p<0.05, according to two ways ANOVA test, followed by Tukey multiple comparison test. *p* values are reported in Supplementary Table 1.

We next assessed the role played by BCG-stimulated BC cells differentially expressing sTn on the first line of innate immunity, by analyzing the macrophage response induced. For that, human macrophages differentiated from the monocytes of healthy blood donors were stimulated for 24 h with the conditioned media from BCG-challenged or unchallenged MCR_Nc_ or MCR_sTn_ cells and the production of various cytokines was measured in their culture medium (Figure 4). After stimulation with conditioned medium from unchallenged MCR_Nc_ or MCR_sTn_ cell lines, macrophages *de novo* produced IL-6 and TNFα, while the basal cytokine secretion of IL-1β and IL-10 remained unchanged (Figure 4 and Supplementary Table 1). Interestingly, when stimulated with the conditioned medium from BCG-challenged MCR_Nc_ or MCR_sTn_ cell lines, macrophages significantly increased secretion of IL-6, IL-1β, TNF-α and IL-10 but not of IL-8 (Figure 4 and Supplementary Table 1). Notably, the induction of cytokine secretion was markedly upregulated when macrophages were stimulated with the conditioned medium from BCG-challenged MCR_sTn_ cells, compared to those stimulated with BCG-challenged MCR_Nc_. The cytokines IL-2, IL-4, IL-12, and IL-17 were never detected in any condition (data not shown).

Collectively, data suggests that the secretome of cells overexpressing sTn, when stimulated with BCG, induce a higher expression of pro-inflammatory cytokines such as IL-6 and TNF-α.

### ST6GALNAC1 overexpression by BC cells promotes down-regulation of genes responsible for maintaining genomic stability

The overall impact of *ST6GALNAC1* expression on the transcriptome of MCR cells was analyzed by expression microarray technology on both MCR_sTn_ and MCR_Nc_ cells.

Considering only genes modulated at least by a log_2_ expression difference ≥ 1.0 (which means a change of at least 2 folds), 488 genes were modulated as a consequence of ST6GALNAC1 overexpression. Of these, 185 displayed up-regulation and 303 down-regulation in MCR_sTn_ compared to MCR_Nc_. The complete list of genes showing significant modulation is reported in Supplementary Table 2.

While several genes were found to be modulated by ST6GALNAC1, we focused our attention on genes with a recognized role in tumor growth and progression, owing the well-known role of sTn in cancer growth. Therefore, we identified 21 genes, whose up- or down-regulation in MCR_sTn_ could, according to literature data, play a putative role in promoting or inhibiting cancer growth. The proportion between cancer genes modulated toward increased or decreased malignancy was 14 to 7, respectively. The cellular functions on which the former genes were involved included apoptosis, cell growth, and angiogenesis, while the cancer-associated genes modulated toward decreased malignancy were involved in cell growth, cytoskeletal rearrangements and proteolytic activity (Supplementary Table 3). In addition, in MCRs_Tn_ cells, a group of 5 genes (which became 12 when a log_2_ expression difference ≥ 0.5 was used, fold change 1.4), collectively referred to as “caretaker genes” due to their role in preserving genomic stability, displayed down-regulation compared to MCR_Nc_ (Table 1 and Supplementary Table 3).

**Table 1:** Genes involved in maintaining chromosomal stability and/or DNA repair showing down-regulation in MCR_sTn_ cells

Genes involvedin preservingchromosomalstability	Fold change	Genes involvedin homologousrecombination repair(HR)	Fold change	Genes involvedin nucleotideexcision repair(NER)	Fold change
*HAUS6*	-2.1	*XRCC4*	-2.1	*ERCC6L*	-2.0
*SGOL1*	-2.0	*BRCA1*	-2.0	*ERCC6*	-1.7
*CSPP1*	-1.6	*XRCC2*	-1.6	*ERCC8*	-1.5
*HAUS3*	-1.5				
*KNTC1*	-1.5				
*CDCA5*	-1.4				

**Figure 5:**
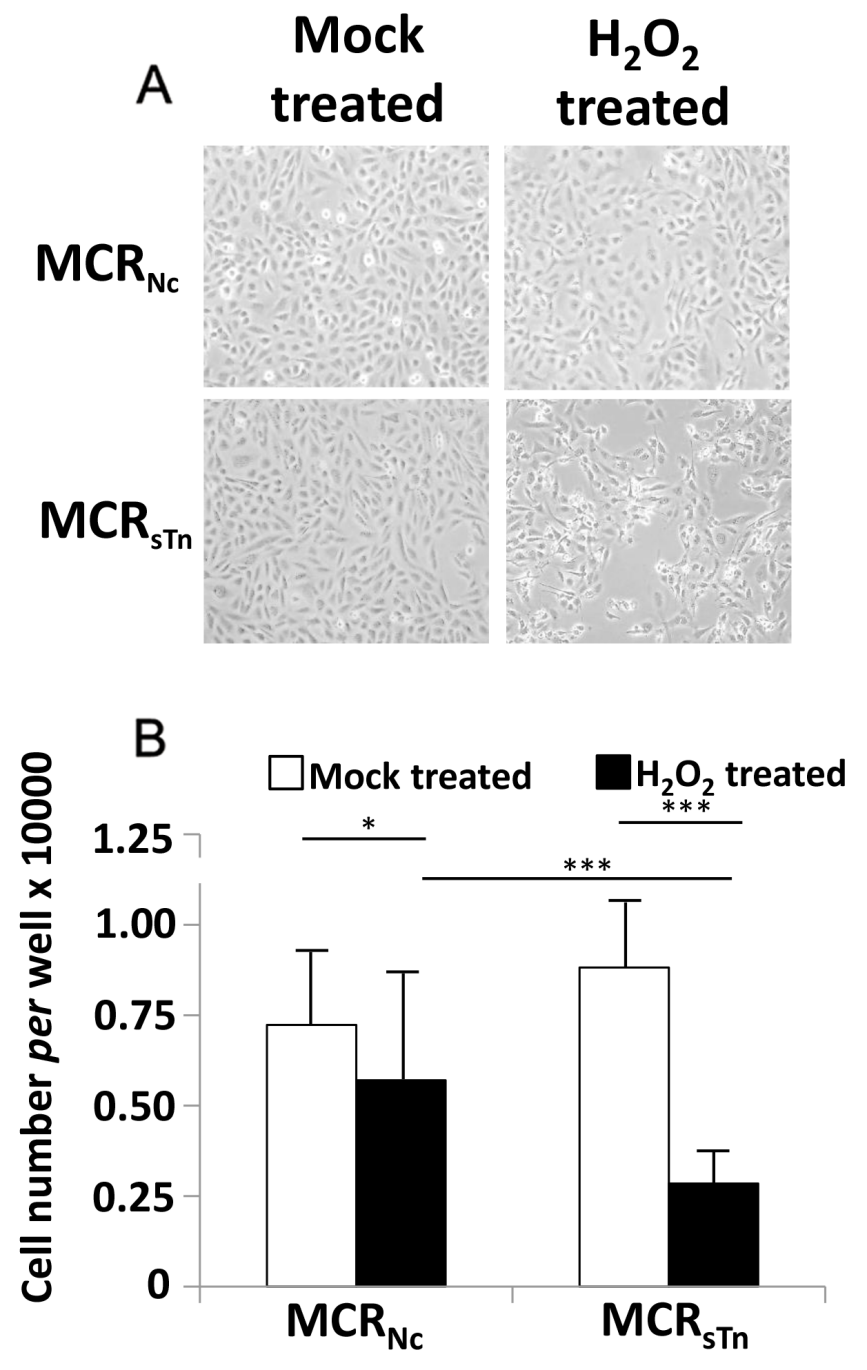
Cytotoxic effect of H_2_O_2_ **(A)** Cells were treated with H_2_O_2_ or mock-treated as described in Materials and Methods and photographed with a phase contrast microscope (original magnification 40 X). **(B)** Cell counting indicated a 20% reduction in MCR_Nc_ cells and a 68% reduction in MCR_sTn_ after H_2_O_2_ treatment. Data are the mean ± SD of at least three experiments performed in triplicate. * *p*< 0.05; *** *p*<0.0001 according to Student's *t* test.

It is expected that the co-ordinate down-regulation of genes involved in preserving genomic stability leads to the exacerbation of genotoxic stress. To obtain a “functional validation” of gene expression data, we compared the sensitivity of the two MCR cell lines to an oxidizing agent, such as H_2_O_2_. As shown in Figure 5, the H_2_O_2_-induced cell death was dramatically higher in MCR_sTn_ than in MCR_Nc_, as shown by phase contrast microscopy (a) and cell counts (b).

### In MCR_sTn_ cells BCG challenging up-regulates genes involved in post-transcriptional regulation

To investigate the differential response to BCG of MCR cells expressing or not the sTn antigen, we analyzed the global gene expression of MCR_sTn_ and MCR_Nc_ after BCG challenge. We classified genes modulated by a log_2_ expression difference ≥ 1.0 (which means a change of at least 2 folds) in broad functional categories (Supplementary Table 4). BCG challenge resulted in the modulation of a few genes which were different in the two MCR cell variants (Table 2 and Supplementary Table 4). The only gene expression change showing parallel modulation in MCR_Nc_ and MCR_sTn_ was the up-regulation of *HLA-DQA2*, encoding the α2 chain of major histocompatibility complex class II DQ. The larger number of genes affected by BCG challenge, but only in MCR_sTn_ cells, was involved in post-transcriptional regulation, including small nucleolar RNA (snoRNA) and small nuclear RNA (snRNA). This suggests that in MCR_sTn_ cells, BCG challenge results in profound alterations of protein expression, in spite of the relatively low number of transcriptionally modulated genes. Other functional categories whose genes were affected only in MCR_sTn_ were within the proteolysis (up-regulation of one gene ≥ 3) and signal transduction category (up-regulation of one gene ≥ 2). The only functional category of genes which were affected only in MCR_Nc_ was the cytoskeleton structure (down-regulation of one gene ≥ 2).

**Table 2:** Modulation of gene expression by BCG challenge

Functional Class	MCR_Nc_	MCR_sTn_
Cell Growth and Survival	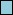 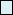 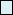	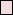 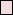
Cytoskeleton Structure	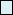	
Inflammatory and Immune Response	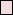 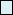	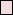
Intracellular Transport	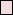 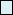	
Postranscriptional Regulation		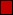 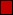 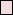 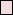 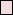 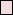 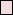 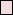 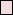
Proteolysis		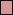
Signal Transduction		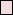
Transcription regulation	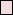	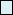
Unclear function		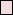 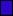

Genes showing a fold change ≥ 2 upon BCG challenging were classified in broad functional categories. Each square represents a gene modulated by BCG according to this code: 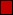
up-regulation ≥ 4 (fold changes); 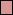
up-regulation ≥ 3, <4; 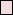
up-regulation ≥ 2, <3; 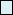
down-regulation ≥ 2, <3; 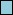
down-regulation ≥ 3, <4; 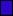
down-regulation ≥ 4.

Seven protein coding genes (*LOX, MME, GLIPR1*, *NR2F1, PLCB1, RANBP2* and *TMPRSS11E*) and one RNA gene (*SCARNA4*) were chosen for validation of the microarray data by RT real time-PCR (Supplementary Figure 1). The two techniques provided consistent results for the seven protein coding genes, while some discrepancies were observed for *SCARNA4*. It is not clear whether this depends on the peculiar nature of this RNA gene and which of the two techniques provides more reliable results.

## DISCUSSION

Response to BCG is typically associated with upregulation of pro-inflammatory cytokines such as IL-6 [19, 20]. In this study we have shown that BC patients responding to BCG-therapy express higher levels of IL-6 mRNAs and that a positive correlation exists between IL-6 and ST6GALNAC1 mRNA, but only among BCG responders. This, together with the fact that in our cohort high ST6GALNAC1 mRNA expressing patients were all BCG responders, provides a basis for the proposed role of sTn as a predictive biomarker for BCG response in BC [10]. To gain further mechanistic insights into the link between STn expression, malignancy and BCG response in BC, we used a BC cell line transduced to overexpress the *ST6GALNAC1* gene as an experimental model. Although the overexpression of ST6GALNAC1 has been widely studied in different cancer cell models and found to be responsible for increased malignancy [21–25], little is known about its role in BC biology [9].

While others have reported that BCG-challenge induces cytokine secretion by BC [1, 20, 26], our results show for the first time that this process is strongly stimulated by ST6GALNAC1/sTn expression. A significantly increased release of IL-8 and IL-6 was observed by the BCG-stimulated MCR cells expressing sTn (MCR_STn_), compared to the negative control cells (MCR_Nc_). These pro-inflammatory cytokines are crucial for the activation and mobilization of immune effectors to the bladder urothelium, being therefore essential for the ultimate eradication of the tumor [27, 28].

Macrophages play a key role in determining the patient's response to BCG therapy. BCG effectiveness against BC cells implies the polarization of macrophages into M1phenotype, which drives the differentiation of helper T cells towards the pro-inflammatory Th1 response, leading to the activation of cytotoxic T cells. The hallmark of M1 is the secretion of pro-inflammatory cytokines, such as IL-6, IL-1β and TNFα and a low secretion of the anti-inflammatory cytokine IL-10. Notably, the macrophages stimulated with conditioned media from BCG-challenged MCR_sTn_ mainly secreted IL-6, IL-8 and TNF-α but very little IL-10, suggesting a phenotype closer to the pro-inflammatory type M1 than to the anti-inflammatory type M2 [29]. This is of particular relevance considering the association of a poor response to BCG with the presence of M2 type macrophages [30]. Moreover, these results provide key information that can explain why the expression of sTn and of sialyl-6-T antigens (the biosynthesis of the former is mainly dependent on *ST6GALNAC1* while that of the latter depends mainly on *ST6GALNAC2*, Figure 1) [11] by BC is predictive of BCG response and is associated with a better recurrence-free survival [10]. On the other hand, MCR_sTn_ cells (not challenged with BCG) and sTn-expressing mucins induce a tolerogenic phenotype in human dendritic cells and in T-lymphocytes, respectively [13, 31]. Together, these data suggest that BCG treatment turns the expression of sTn/sialyl-6-T from an unfavorable condition associated with increased progression and immune evasion into a favorable prognostic marker of a better response to BCG therapy.

The link between STn expression and malignancy in bladder cancer was further investigated through an exhaustive analysis of the *ST6GALNAC1*-induced transcriptome modulation *via* gene expression microarray technology. We observed a prevalence of changes oriented toward increased malignancy in MCR_sTn_, including modulation of genes controlling cell growth and/or apoptosis, which is consistent with the reported increased proliferation rate of MCR_sTn_ [9]. However, the most prominent transcriptomic change induced by the constitutive overexpression of *ST6GALNAC1* in MCR cells was the decreased expression of genes involved in maintaining genomic stability. Consistently, we observed a dramatically higher sensitivity to H_2_O_2_-induced cell damage of MCR_sTn_ in comparison with MCR_Nc_. This is of particular interest if one considers that the generation of reactive oxygen species by BCG is a crucial mechanism of BCG-induced damage to BC cells [4]. Thus, it is possible that an increased sensitivity to oxidizing agents by sTn-expressing bladder cancers is one of the basis of their better response to BCG therapy.

Although the secretion of cytokines IL-6 and IL-8 displayed a marked increase in MCR cells after BCG challenge, we failed to observe a consistent up-regulation of their genes. As a possible explanation, it should be considered that in some circumstances the secretion of cytokines is regulated by the release of preformed molecules stored in vesicles, rather than by *de novo* transcription of their genes [32].

The challenge with BCG modulated a higher number of genes in MCR_sTn_ cells than in MCR_Nc_ cells, suggesting that the presence of the sTn antigen on membrane glycoproteins affects the development of the genetic program triggered by BCG contact. A low number of protein coding genes were modulated by BCG, while a high number of non-coding RNA genes were up-regulated, but only in the MCR_sTn_ variant. The majority of these RNA genes belonged to the group of small nucleolar RNAs which guide the methylation or pseudouridylation of other RNAs [33, 34], regulating protein expression. It is possible that through these non-coding RNAs, BCG has a higher ability to modulate the proteome of sTn-expressing BC cells, including secretion of cytokines.

Although the results we obtained with this cell line should be generalized with great caution, we have shown that in BC cells sTn expression can induce changes of gene expression putatively associated with increased malignancy. Furthermore, the down-regulation of genes responsible for genomic stability results in increased sensitivity to oxidizing agents, like those produced by BCG infection. This, together with the induction of an increased inflammatory response by macrophages, can explain why the expression of the sTn antigen by BC cells can be a predictive marker for a successful BCG therapy.

## MATERIALS AND METHODS

### Surgical specimens

Matched pairs of histologically verified non-muscle-invasive bladder cancers (multiple tumors primary or recurrent) and normal appearing mucosa remote from the tumor were collected from 43 patients, at Hospital São José (Lisbon, Portugal). Tissue samples were immediately immersed in RNAlater® RNA Stabilization Reagent (Sigma), stored at 4°C, overnight and then preserved until further processing at -20°C. Patients were treated with transurethral resection of the bladder tumors (TURBT) followed by BCG instillations (TICE® BCG). None of these patients had received previous adjuvant therapy. Prior patient consent and approval from the institute research ethics committee were obtained. According to recurrence, we named “BCG responders” those patients without recurrence at least until one year after TURBT (29 patients) and “BCG non-responders” those experiencing recurrence within one year after TURBT (14 patients).

### ST6GALNAC1-expressing MCR cells

The cell line MCR, obtained from a subcutaneous metastatic lesion of an invasive transitional cell cancer of the bladder [35], was grown in DMEM (4.5 g/L glucose), containing 10% Foetal Calf Serum (FCS), 2 mM *L*-glutamine and 100 μg/mL penicillin/streptomycin, all from Sigma. Transduction with a ST6GALNAC1-expressing lentiviral vector was described elsewhere [9]. The transduced MCR cells were enriched in their sTn positive population (MCR_sTn_), by magnetic-activated cell sorting as recommended by the manufacturer (Miltenyi Biotec), using anti-sTn antibody (HB-STn1 clone, Dako).

### Real time RT-PCR

Total RNA was isolated using the GenElute Mammalian Total RNA Purification kit (Sigma), according to the manufacturer's instructions. One microgram of total RNA was reverse transcribed using the random-primers based High Capacity cDNA Archive Kit (Applied Biosystems). The expression level of *ST6GALNAC1* (Hs00300842_m1) *IL-6* (Hs00174131_m1), *LOX* (Hs00942480_m1), *MME* (Hs00153510_m1), *GLIPR1* (Hs01564142_m1), *NRF2* (Hs01354342_mH), *SCARNA4* (Hs03298714_s1), *PLCB1* (Hs01001930_m1), *RANBP2* (Hs00196669_m1) and *TMPRSS11E* (Hs01070171_m1) was evaluated with the TaqMan assay system in a 7500 Fast Real-Time PCR System (Applied Biosystems) using the TaqMan Universal PCR Master Mix Fast, as described previously [13, 36, 37]. The efficiency of the amplification reaction for each primer-probe was above 95% (as determined by the manufacturer). Normalized mRNA expression was computed as number of mRNA molecules of the gene of interest per 1000 mRNA molecules of the endogenous control β-actin gene, calculated using the 2^-ΔCT^×1000 formula [38].

### BCG challenge of MCR cells

Commercial Connaught BCG (ImmuCyst, Sanofi Pasteur SA, France) was suspended in PBS containing 0.05% Tween 80 and stored at -80 °C. Before each assay, BCG aggregates were discarded by centrifugation (300 × g for 5 min). To assess BCG internalization, bacteria were stained with 2 μg/mL of 5-(and-6-)(((4-chloromethyl)benzoyl)amino)tetramethylrhodamine (CMTR, Invitrogen) for 2 h in culture medium, incubated with MCR_Nc_ or MCR_sTn_ cells in a 1:10 cell/bacteria ratio for 2 h at 37 °C and analyzed by flow cytometry. To assess cytokine secretion, MCR_Nc_ or MCR_sTn_ cells were challenged with unstained BCG for 2 h at 37 °C, the medium was removed and the cells were washed twice with PBS and incubated with fresh medium for 16 h. Conditioned media were used for cytokine analysis and to challenge macrophages, while cell pellets were used for RNA extraction and transcriptomic analysis.

### Determination of cytokine concentration

The concentration of cytokines IL-1β, IL-2, IL-4, IL-6, IL-8, IL-10, IL-12, IL-17, IFNγ and TNFα was measured in a 96-well strip plate from a commercial MIBA kit (Bio-Rad), as recommended by manufacturer's instructions. Fluorescence was read in a Luminex 100 Bio-Plex Liquid Array Multiplexing System reader (Bio-Rad) and the data analyzed with the Bio-Plex Manager v5 software (Bio-Rad).

### Macrophage preparation and stimulation

Mononuclear cells were isolated by Ficoll-Hypaque density gradient centrifugation (GE Healthcare) from the peripheral blood of healthy blood-donors, obtained from the Blood Collection Service of the Pizzardi Hospital of Bologna, according to the requirements of the local ethical committee. Macrophages were obtained by differentiation of monocytes by culture in RPMI 1640 (Sigma) medium supplemented with 20% FCS, 2 mM L-glutamine and 100 μg/mL penicillin/streptomycin. After 7 days, monocyte-derived macrophages were detached with a cell scraper and dispensed in 24 well plates at a cell density corresponding approximately to 50% of confluence. One day later, macrophages were incubated with standard unconditioned culture medium or with the media conditioned by MCR_Nc_ or MCR_sTn_ cells either BCG-challenged (as described above) or mock challenged. After 2 h, the conditioned media were replaced by fresh medium, which was collected 24 h later and stored at -80 °C for the detection of cytokines secreted by macrophages.

### Whole transcriptome analysis by expression microarray

Total RNA was isolated by the guanidinium thiocyanate-method [39] and converted to labelled single strand cDNA (ssDNA) by the commercial Whole Transcript Expression kit (Ambion), according to the manufacturer's instructions. Labelled ssDNA fragments were hybridized in a Human Transcriptome Array 2.0 overnight. After staining with phycoerythrin-streptavidin, fluorescence was read in a GeneChip Scanner 3000 7G (Affymetrix). Raw data were background-subtracted, normalized and summarized with the robust multi-array average (RMA) algorithm implemented in the Affy package of Bioconductor (http://www.bioconductor.org). Differentially expressed genes between query and control assay were selected by application of the *t*-test with a p ≤ 0.05 cut-off and by the log_2_ expression ratio, considering only variations ≥0.5. Array data were analyzed by the ArrayStar v2.0 software (DNASTAR) and through a literature search of the biological roles of modulated genes. Gene nomenclature followed the *HUGO Gene Nomenclature Committee* rules (http://www.genenames.org/) in italic uppercase letters. With exception of cytokines, proteins had the same name as the gene, represented in regular uppercase.

### H_2_O_2_ treatment

Cells were incubated for 1 h in serum-free medium without H_2_O_2_ (control treatment) or with 5 mM H_2_O_2_. Cells were then rinsed, grown for 24 h in complete medium, harvested, stained with trypan blue and counted.

## SUPPLEMENTARY MATERIALS FIGURES AND TABLES









## Abbreviations

BC: bladder cancer
BCG: *Bacillus Calmette-Guérin*
CMTR: 5-(and-6)-(((4-chloromethyl)benzoyl)amino)tetramethylrhodamine
DMEM: Dulbecco's modified Eagle medium
FCS: foetal calf serum
Gal: Galactose
GalNAc: N-acetylgalactosamine
GlcNAc: N-acetylglucosamine
MIBA: multiplex immune-beads assay
PE: phycoerythrin
RPMI: Roswell Park Memorial Institute
RT-PCR: reverse transcriptase-polymerase chain reaction
Sia: sialic acid
sTn: sialyl-Tn
TURBT: transurethral resection of the bladder tumors.
