# Supplementary material for: Expression of sialyl-Tn sugar antigen in bladder cancer cells affects response to Bacillus Calmette Guérin (BCG) and to oxidative damage
Source: Oncotarget. 2017 Apr 17;8(33):54506–17. doi: 10.18632/oncotarget.17138 (PMC5589598; doi:10.18632/oncotarget.17138)
Supplement: Supplementary file 1 [file oncotarget-08-54506-s001.pdf]

Expression of sialyl-Tn sugar antigen in bladder cancer cells affects response to *Bacillus Calmette Guérin* (BCG) and to oxidative damage

SUPPLEMENTARY FIGURE AND TABLES

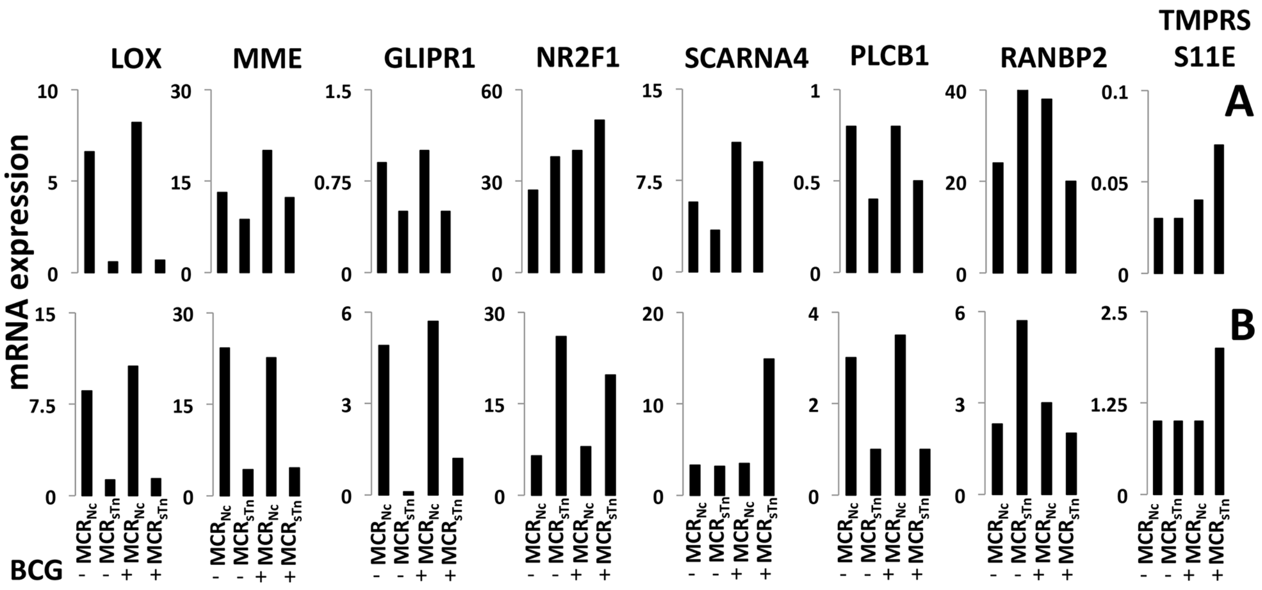

Supplementary Figure 1: mRNA quantification by real time RT-PCR (A, upper histograms) or microarray analysis (B, lower histograms). RNA was from MCR_{Nc} or MCR_{sTn}, challenged or not with BCG (indicated at the bottom). The RNA level determined by real time RT-PCR is expressed as %0 of β-actin molecules, while the RNA level determined by microarray was expressed in arbitrary units calculated as: RNA expression= $2^{(log2E-5)}$ Where 5 is the log2E of a gene not expressed.

Supplementary Table 1: *p* values calculated with ANOVA, followed by Tukey multiple comparison test for data reported in Figure 3 and Figure 4.

***p* values for data of Figure 3**		
**Conditioned media of MCR cells**	***p* values**	
	**IL-8**	**IL-6**
MCR_{Nc} unchall. vs MCR_{sTn} unchall.	0.91	0.42
MCR_{Nc} BCG-chall. vs MCR_{sTn} BCG-chall.	0.05	<0.0001
MCR_{Nc} unchall. vs MCR_{Nc} BCG-chall.	0.91	<0.01
MCR_{sTn} unchall. vs MCR_{sTn} BCG-chall.	0.05	<0.0001
Interaction	0.14	<0.0001

***p* values for data of Figure 4**					
**Conditioned media of MØ stimulated with:**	***p* values**				
	**IL-8**	**IL-6**	**IL-1β**	**TNF-α**	**IL-10**
MCR_{Nc} unchall. vs MCR_{sTn} unchall.	0.51	0.9	1	0.9	1
MCR_{Nc} BCG-chall. vs MCR_{sTn} BCG-chall.	0.67	0.19	<0.01	0.08	0.12
MCR_{Nc} unchall. vs MCR_{Nc} BCG-chall.	0.41	0.2	<0.01	<0.05	0.1
MCR_{sTn} unchall. vs MCR_{sTn} BCG-chall.	0.77	<0.05	<0.0001	<0.01	<0.01
Interaction	0.1	0.31	<0.01	0.16	0.13

Supplementary Table 2: Genes modulated in MCR_{sTn} cells as compared to MCR_{Nc} cells.

See **Supplementary File 1**

Supplementary Table 3: Cancer-associated genes modulated in MCR_{sTn} cells as compared to MCR_{Nc} cells.

See **Supplementary File 2**

Supplementary Table 4: Genes modulated by BCG in MCR_{Nc} and/or MCR_{sTn}.

See **Supplementary File 3**
